# Association of One-Carbon Metabolism-Related Vitamins (Folate, B6, B12), Homocysteine and Methionine With the Risk of Lung Cancer: Systematic Review and Meta-Analysis

**DOI:** 10.3389/fonc.2018.00493

**Published:** 2018-10-31

**Authors:** Jia Yang, Hongjia Li, Haibin Deng, Zhongqi Wang

**Affiliations:** ^1^Oncology Department of LongHua Hospital, Shanghai University of Traditional Chinese Medicine, Shanghai, China; ^2^Shanghai University of Traditional Chinese Medicine, Shanghai, China

**Keywords:** one-carbon metabolism, folate (vitamin B9), vitamin B6, lung cancer, serum, meta-analysis

## Abstract

**Background:** Studies on serum one-carbon metabolism factors (folate, B6, B12, homocysteine, and methionine) with lung cancer (LC) risk have produced inconsistent results. We aimed to systematically evaluate the association between them.

**Methods:** This study was reported in accordance with the PRISMA Statement and was registered with PROSPERO (no. CRD42018086654). Relevant studies were searched in PubMed, Embase, MEDLINE, and CNKI up to February 2018. Random-effects models were used to estimate the pooled standardized mean differences (SMD) or odds ratios (OR), as well as their 95% confidence interval (CI). Sensitivity and subgroup analysis were performed to identify the source of heterogeneity. Publication bias was also assessed.

**Results:** A total of 14 articles (8,097 patients) were included. The concentration of serum folate and vitamin B6 of LC patients were lower than the controls [SMD −0.53, 95% CI (−0.70, −0.35), *p* = 0.001 and SMD −0.28, 95%CI (−0.53, −0.02), *p* = 0.001, respectively]. While the concentration of homocysteine of the cases was higher than the controls [SMD 0.41, 95% CI (0.24, 0.59), *p* = 0.001]. However, there were no significant differences between LC patients and the controls in terms of vitamin B12 and methionine [SMD −0.09, 95% CI (−0.27, 0.09), *p* = 0.202 and SMD −0.13, 95% CI (−0.36, 0.10), *p* = 0.001]. Subgroup analysis showed that these results were more significant in Europe, Asia, former and current smokers, and the male population (*p*-value < 0.05).

**Conclusions:** Serum folate and vitamin B6 might be protective factors against lung carcinogenesis and homocysteine could contribute to LC risk.

## Introduction

Despite advancement in treatment, lung cancer remains the most common and fatal cancer in the world (1). At present, 60% of lung cancer cases are in an advanced stage or metastatic at the time of diagnosis, highlighting the importance of finding serum biological variables in early stage of lung cancer (LC) (2).

A growing body of evidence shows that DNA methylation plays an important role in the progression of cancers (3). DNA methylation requires a methyl donor, which is mainly provided by one-carbon metabolism (OCM) (4, 5). S-adenosylmethionine (SAM), the activated form of methionine, serves as the most important methyl donor. It can disrupt methionine cycle and change cytosine methylation in DNA by reducing intracellular SAM levels. In fact, the OCM involves a variety of factors, including folate (vitamin B9), vitamin B6, vitamin B12, homocysteine, and methionine. These factors interact with each other in a complex biochemical metabolic pathway. Through the mediating effects of these factors, one-carbon groups are transferred to maintain DNA methylation, regulate gene synthesis, and provide important material basis for biological functions (6, 7). Moreover, OCM is a dynamic process, in which the decrease or accumulation of one component can affect DNA methylation and genomic integrity, resulting in altered epigenetic modification, imbalanced of tumor suppressor genes and oncogene and induced malignant transformation (8).

However, researchers are still debating on the role of these factors in the development of LC. Some studies found no association between some OCM related factors and LC risk (9–12). However, increasing numbers of evidence have shown that B vitamins and methionine were correlated with LC risk, not only in dietary supplements (13–17).

To the best of our knowledge, previous meta-analyses have not analyzed the relationship between all OCM factors in serum and LC risk. Thus, this study aims to systematically and comprehensively estimate the association of B vitamins (folate, B6, B12), homocysteine and methionine with LC risk in order to evaluate their important roles in LC.

## Methods

This systematic review and meta-analysis was reported in accordance with the Preferred Reporting Items for Systematic Reviews and Meta-Analysis (PRISMA) Statement (additional file: Checklist S1) and was registered with PROSPERO (no. CRD42018086654).

### Search

We systematically searched the PubMed, Embase, MEDLINE and CNKI (China National Knowledge Infrastructure) databases to identify relevant studies up to February 1, 2018. The search, which placed no restrictions on language or publication status, used the following search terms: (Lung Neoplasms [MeSH]) AND ((Vitamin B9 [MeSH] OR Folate [MeSH]) OR (Vitamin B6 [MeSH] OR Pyridoxal 5-Phosphate [MeSH]) OR (Vitamin B12 [MeSH] OR Cyanocobalamin [MeSH]) OR Homocysteine [MeSH] OR Methionine [MeSH]). We also reviewed the relevant references cited in the retrieved articles. The search strategy used for PubMed is listed in Appendix A (Supplementary Material).

### Selection criteria

Studies were included in the meta-analysis if they met the following criteria: (1) the study focused on the association between serum B vitamins (folate, B6, B12) or homocysteine or methionine involved in one-carbon metabolism and lung cancer risk; (2) the outcome was lung cancer incidence; (3) case-control study, cohort study, or randomized controlled trial (RCT); and (4) the variables were reported as means (M) and standard deviations (SD), or as odds ratio (OR) and 95% confidence intervals (95% CI).

The exclusion criteria were (1) studies on dietary intake or supplementation of these B vitamins, homocysteine and methionine; (2) overlapping articles or studies that involved overlapping data; (3) conference abstract or meta-analyses; and (4) studies lacking healthy controls. If the same data were presented in more than one study, we included the study with the largest number of cases or provided more detailed information.

### Data extraction

Two investigators independently extracted the following data: first author, publication year, region/country, methods, study design, sample size, age, gender, source of controls and smoking status. We also extracted means (M) and standard deviations (SD) for the serum levels of B vitamins (folate, B6, B12), homocysteine and methionine as well as odds ratio (OR) and 95% confidence interval (CI). To ensure the accuracy of the data, inconsistencies were discussed with another reviewer until a consensus was reached.

### Quality assessment

The Newcastle-Ottawa Scale (NOS) (18) was used to assess the methodological quality of the included studies (Table S1). The NOS consists of 3 aspects: selection, comparability, and exposure in the primary study. The total score ranged from 0 to 9 stars. Studies with 6 or more stars were classified as high-quality studies; studies with fewer than 6 stars were considered to be of low quality (19).

### Data analysis

All reported *P*-values were 2-sided; *P-*values < 0.05 were considered statistically significant. Standardized mean difference (SMD) and 95% confidence interval (95% CI) were used to estimate the serum folate, B6, B12, homocysteine and methionine in the overall and subgroup (by region, method) population; Odds ratio (OR) and 95% CI were used to evaluate these factors in subgroup population (by gender, smoking status). If the pooled effect size SMD < 0 or OR < 1, the factor was protective; otherwise it was a risk factor.

Heterogeneity was calculated by Q test (*P*_*het*_) and *I*-squared (*I*^2^) statistics test. If *P*_*het*_ < 0.1 or *I*^2^ > 30%, then there was significant heterogeneity. The fixed-effect model was used if there was no heterogeneity; otherwise, a random-effect model was applied. If significant heterogeneity existed, sensitivity and subgroup analysis was used to explore heterogeneity sources and studies with 95%CI markedly deviated from the others or factors leading to *I*^2^ largely decreased were primary originators.

Publication bias were assessed using funnel plots and Egger test when the number of studies for biological variable was more than ten (20). If *P* < 0.1, publication bias exited and then a trim-and-fill method was implemented to estimate the effect of publication bias on the results (21). All statistical analyses were performed using STATA version 12.0 software (Stata Corp LP, College Station, TX, USA).

## Results

### Literature search and study characteristics

**Figure 1:**
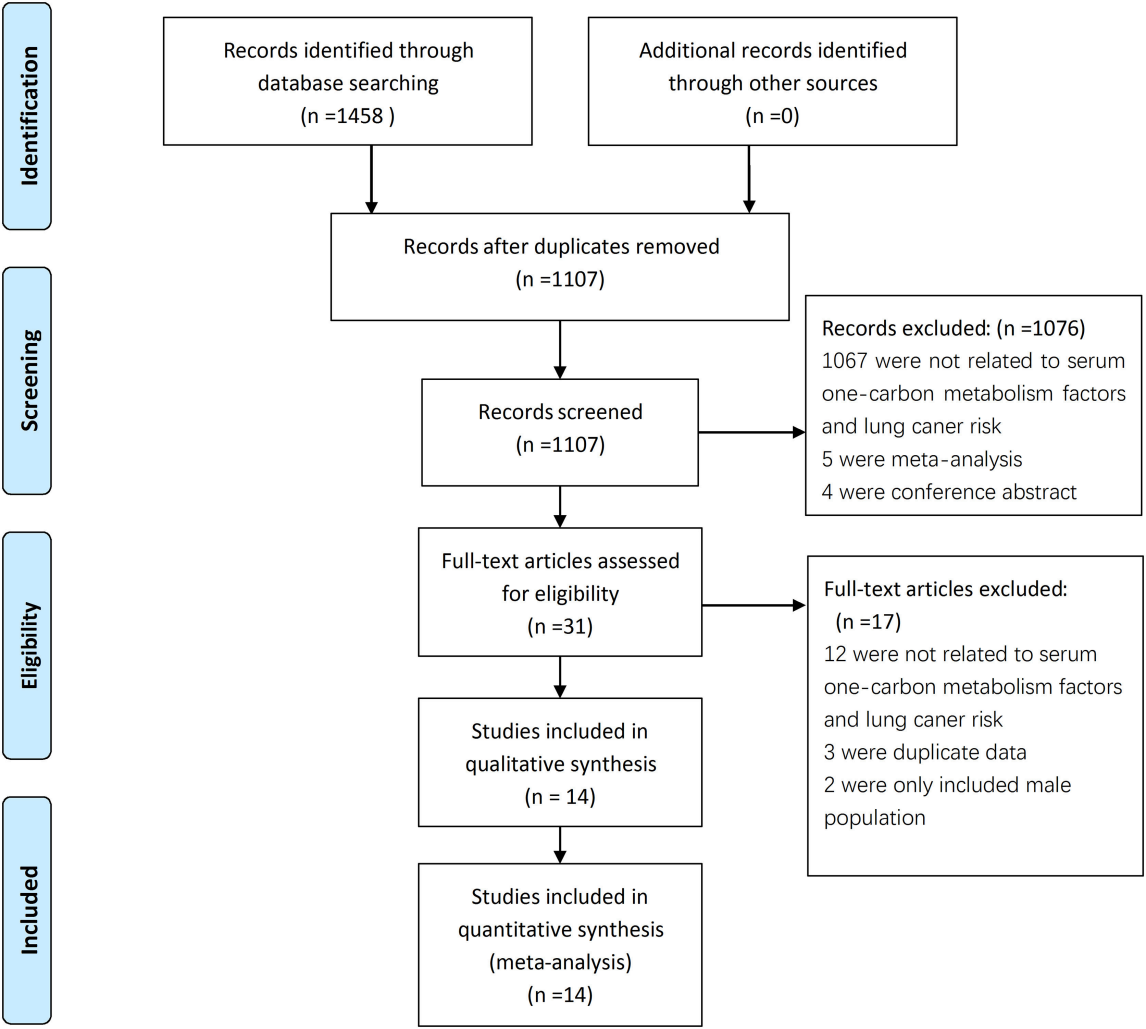
Flow chart of the study selection process.

A total of 1,458 articles were retrieved from the literature search, and 1,107 articles were obtained after the removal of duplicates. After the titles and abstracts read, 765 articles were found unrelated to the serum factors with lung cancer and were excluded. Then excluded were 302 studies about oral supplements, 4 conference abstracts, and 5 meta-analyses; after this exclusion, 31 articles remained. After careful reading of the full texts of these articles, 17 articles were further excluded from the study because 12 provided no information on relationship between serum factors and LC risk, 2 only included male population (22, 23), 3 were duplicate data (16, 24, 25). Thus, 14 articles (17, 22, 23, 26–38) were finally included into this systematic review and meta-analysis; The studies included 8,097 lung cancer patients and 10,008 healthy controls (Figure 1). Four studies were from Europe, nine were from Asia, and one was conducted in Europe, Asia, and America. Eleven of the studies were case-control studies, and three were nested case-control studies. The main features of these 14 studies are shown in Table 1.

**Table 1:** Characteristics of 14 studies included in the meta-analysis.

**Study**	**Sample size (cases/controls)**	**Variable**	**Study design**	**Country**	**Region**	**Methods**	**Male (%)**		**Smoking status (%) (Never/Former/Current)**		**NOS Score^a^**
							**Cases**	**Controls**	**Cases**	**Controls**
Durda et al. (27)	366/366	Folate	Case-control	Poland	Europe	HPLC	62.8	62.8	37.2/NA/63.8	38.5/NA/61.5	8
Fanidi et al. (17)	5,364/5,364	Folate, vitamin B6, methionine	Nested case-control	United States, Australia, Italian, Greek, Sweden, Finland, Norway, China, Singapore,	North America, Europe, Australia, Asia	North America, Europe, Australia, Asia	54.2	54.2	24.7/28.3/47.0	24.7/28.3/47.0	9
Yu (34)	203/335	Homocysteine	Case-control	China	Asia	HPLC	70	60	NA	NA	5
Baltar et al. (28)	891/1,747	Folate, vitamin B6, vitamin B12, homocysteine, methionine	Nested case-control	Great Britain, Germany, Italy, Spain, The Netherlands, Greece, Sweden, France	Europe	HPLC, Microbiological	62	62	NA	NA	7
Johansson et al. (26)	899/1,815	Vitamin B6, folate, vitamin B12, homocysteine, methionine	Nested case-control	Great Britain, Germany, Italy, Spain, The Netherlands, Greece, Sweden, France	Europe	HPLC, Microbiological	62	62	11/29/59	39/37/23	8
Wei et al. (36)	42/60	Folate, vitamin B12, homocysteine	Case-control	China	Asia	CLIA	60	58	NA	NA	5
Xu (35)	46/35	Folate, vitamin B12, homocysteine	Case-control	China	Asia	CLIA	56.5	57.1	NA	NA	6
Yu (37)	35/30	Folate, vitamin B12, homocysteine	CASE-control	China	Asia	CLIA	60	53.3	NA	NA	6
Zhu et al. (38)	22/50	Folate, homocysteine	Case-control	China	Asia	RIA, HPLC	51	46	NA	NA	4
Mei (33)	30/50	Folate	case-control	China	Asia	RIA	60	56	NA	NA	6
Ozakan et al. (30)	37/26	Folate, homocysteine	Case-control	Turkey	Asia	RIA, HPLC	86.5	80.8	11/22/67	19/50/31	4
Jatoi et al. (29)	46/44	Folate, homocysteine	Case-control	New England	Europe	RIA, HPLC	80	80	10/90/NA	20/80/NA	6
Taso et al. (31)	50/16	Folate, vitamin B6, vitamin B12	Case-control	Taiwan	Asia	HPLC, RIA	52	50	100/NA/NA	100/NA/NA	5
Hu et al. (32)	66/70	Folate	Case-control	China	Asia	CLIA	81.8	65.7	NA	NA	4

a *Study quality was judged based on the Newcastle-Ottawa Scale (range, 1–9 stars)*.

*NA, not available; HB, hospital based; PB, population based; BMI, body mass index; PLP, pyridoxal 5′-phosphate; HPLC, high performance liquid chromatography; RIA, radioimmunoassay; CLIA, chemiluminescence immunoassay; Microbiological, Lactobacillus casei/Lactobacillus leichmannii*.

### Quality assessment (the NOS score)

The result of quality assessment of the included studies based on Newcastle-Ottawa Scale (NOS) was summarized in Table 2. According to the quality evaluation criteria, these 14 studies scored from 4 to 9, and Eight of them were high-scoring studies (quality score ≥ 6).

**Table 2:** Quality assessment of included studies based on the Newcastle–Ottawa Scale for assessing the quality of case control studies.

**Study**	**Selection (score)**				**Comparability (score)**		**Exposure (score)**			**Total score^a^**
	**Definition the case adequate**	**Representativeness of the cases**	**Selection of Controls**	**Definition of Controls**	**Based on the design or analysis**	**Additional factor for study controls**	**Ascertainment of exposure**	**Same ascertainment method**	**Non-Response rate**
Durda et al., (27)	1	1	1	1	1	1	0	1	1	8
Fanidi et al., (17)	1	1	1	1	1	1	1	1	1	9
Yu, (34)	1	1	0	1	1	0	0	1	0	5
Baltar et al., (28)	1	1	1	1	1	0	0	1	1	7
Johansson et al., (26)	1	1	1	1	1	1	0	1	1	8
Wei et al., (36)	1	1	0	1	1	0	0	1	0	5
Xu, (35)	1	1	1	1	0	1	0	1	0	6
Yu, (37)	0	1	1	1	1	1	0	1	0	6
Zhu et al., (38)	0	1	1	1	0	0	0	1	0	4
Mei, (33)	1	1	1	1	1	0	0	1	0	6
Ozakan et al., (30)	0	1	0	1	1	0	0	1	0	4
Jatoi et al., (29)	1	1	0	1	1	1	0	1	0	6
Taso et al., (31)	1	1	0	1	1	0	0	1	0	5
Hu et al., (32)	1	1	0	1	0	0	0	1	0	4

*A study can be awarded a maximum of one star for each numbered item within the Selection and Exposure categories. A maximum of two stars can be given for comparability*.

a *Total score was calculated by adding up the points awarded in each item*.

### Meta-analysis

#### Folate (vitamin B9)

**Figure 2:**
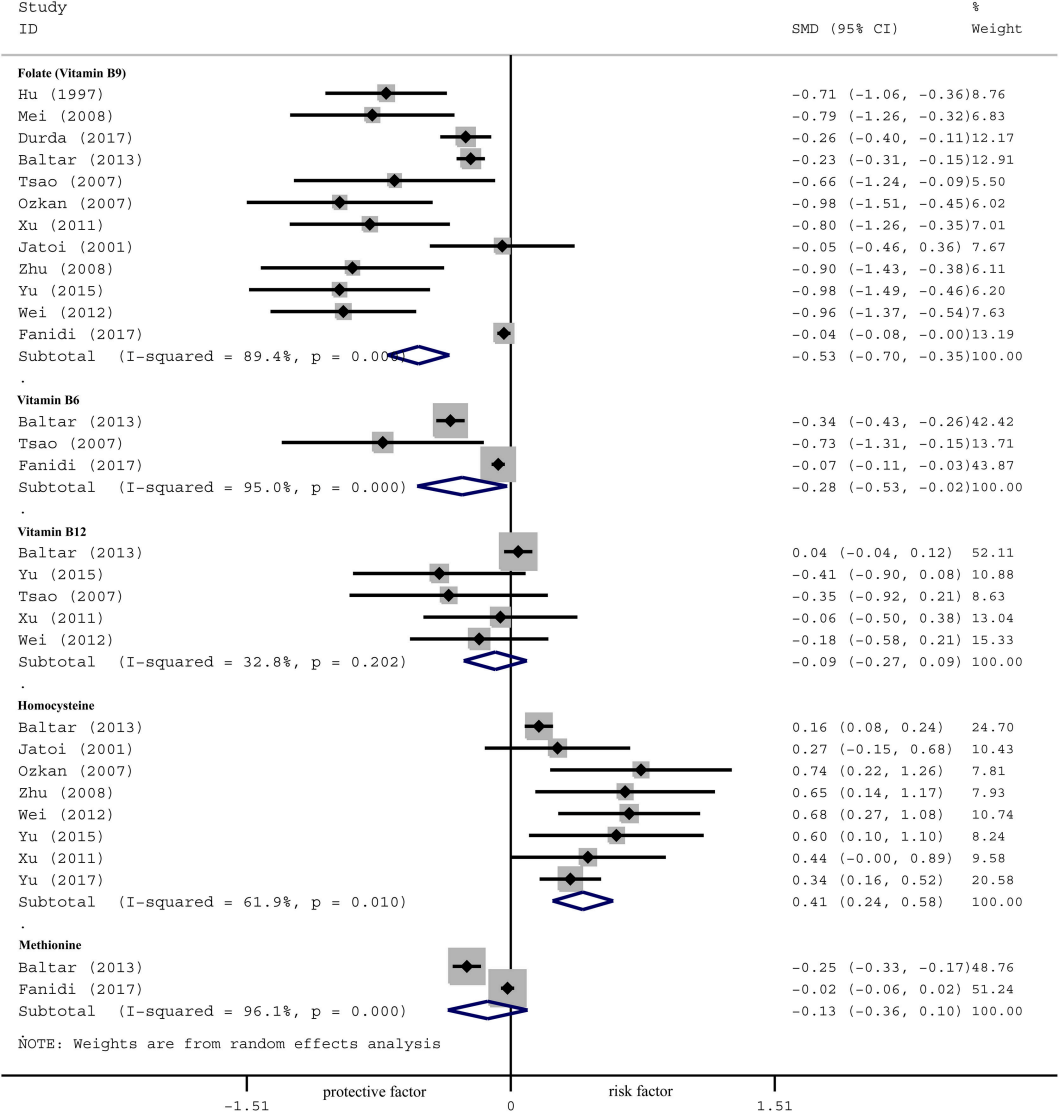
Forest plot for OCM-related factors (folate, vitamin B6, vitamin B12, homocysteine, and methionine) and the risk of lung cancer.

The concentration of serum folate of LC patients was significantly lower than the controls in the overall population, as shown in Figure 2 [SMD −0.53, 95% CI (−0.70, −0.35), *p* = 0.001]. Further subgroup analyses were carried out according to clinical features. By region, serum folate level of LC patients was lower in Europe [SMD −0.23, 95%CI (−0.30, −0.16), *p* = 0.001] and Asia [SMD −0.84, 95%CI (−1.01, −0.67), *p* = 0.001], but not in the US [SMD −0.02, 95%CI (−0.08, 0.03), *p* = 0.435]. By method, serum folate level was lower in using CLIA/RIA [SMD −0.74, 95%CI (−0.95, −0.54), *p* = 0.001] and HPLC method [SMD −0.26, 95%CI (−0.40, −0.11), *p* = 0.001], but not by microbiological method [SMD −0.13, 95%CI (−0.32, 0.06), *p* = 0.168]. By gender, serum folate level was lower in male patients [OR 0.82, 95%CI (0.73, 0.92), *p* = 0.001], but not in females [OR 0.94, 95%CI (0.84, 1.05), *p* = 0.272]. By smoking status, the difference was especially significant in former smokers [OR 0.70, 95%CI (0.62, 0.79), *p* = 0.001] and current smokers [OR 0.86, 95%CI (0.75, 0.99), *p* = 0.030], but was not significant in never smokers [OR 0.86, 95%CI (0.75, 1.0), *p* = 0.052] (Table 3).

**Table 3:** Subgroup analyses of serum OCM-related factors (folate, vitamin B6, vitamin B12, homocysteine, and methionine) associated with lung cancer risk.

		By region*			By method*			By gender**		By smoking status**		
		**Europe**	**Asia**	**US**	**CLIA/ RIA**	**HPLC**	**Biological**	**Men**	**Women**	**Never smokers**	**Former smokers**	**Current smokers**
											
Folate (nmol/L) (*N* = 6,995/7,896)	ES	−**0.23**	−**0.84**	−0.02	−**0.74**	−**0.26**	−0.13	**0.82**	0.94	0.86	**0.7**	**0.86**
	95% CI	−**0.30**, −**0.16**	−**1.01**, −**0.67**	−0.08, 0.03	−**0.95**, −**0.54**	−**0.40**, −**0.11**	−0.32, 0.06	**0.73, 0.92**	0.84, 1.05	0.75, 1.00	**0.62, 0.79**	**0.75, 0.99**
	*P*^a^	**0.001**	**0.001**	0.435	**0.001**	**0.001**	0.168	**0.001**	0.272	0.052	**0.001**	**0.03**
	*I*^2^	**0.00%**	**0.00%**	NA	42.60%	NA	94.20%	**25.90%**	NA	**36.70%**	**32.80%**	**27.90%**
	*P*${}_{\text{het}}^{\text{b}}$	0.451	0.324	NA	0.041	NA	0.006	0.026	NA	0.112	0.071	0.207
Vitamin B6 (nmol/L) (*N* = 6,305/7,195)	ES	−**0.32**	−**0.38**	−0.04	NA	NA	NA	**0.7**	1.08	0.95	**0.68**	**0.76**
	95% CI	−**0.41**, −**0.23**	−**0.54**, −**0.23**	−0.09, 0.02	NA	NA	NA	**0.56, 0.87**	0.98, 1.21	0.36, 2.48	**0.59, 0.78**	**0.62, 0.95**
	*P*^a^	**0.001**	**0.001**	0.207	NA	NA	NA	**0.002**	0.152	0.91	**0.001**	**0.013**
	*I*^2^	**12.50%**	**10.20%**	NA	NA	NA	NA	58.60%	NA	95.10%	21.60%	66.20%
	*P*${}_{\text{het}}^{\text{b}}$	0.572	0.364	NA	NA	NA	NA	0.016	NA	0.045	0.012	0.016
Vitamin B12 (pmol/L) (*N* = 1,064/1,956)	ES	0.04	−0.23	NA	−0.23	NA	0.04	NA	NA	0.85	1.15	1.21
	95% CI	−0.04, 0.12	−0.46, 0.01	NA	−0.46, 0.01	NA	−0.04, 0.12	NA	NA	0.58, 1.25	0.86, 1.53	0.91, 1.61
	*P*^a^	0.304	0.055	NA	0.055	NA	0.304	NA	NA	0.42	0.35	0.19
	*I*^2^	**0.00%**	**0.00%**	NA	**0.00%**	NA	**0.00%**	NA	NA	NA	NA	NA
	*P*${}_{\text{het}}^{\text{b}}$	0.165	0.173	NA	0.145	NA	0.234	NA	NA	NA	NA	NA
Homocysteine (μmo l/L) (*N* = 1,330/2,395)	ES	**0.16**	**0.45**	NA	**0.58**	**0.34**	NA	NA	NA	0.78	0.83	1.05
	95% CI	**0.09, 0.24**	**0.32, 0.59**	NA	**0.32, 0.84**	**0.15, 0.52**	NA	NA	NA	0.58, 1.07	0.64, 1.29	0.81, 1.36
	*P*^a^	**0.001**	**0.001**	NA	**0.001**	**0.001**	NA	NA	NA	0.38	0.177	0.71
	*I*^2^	**0.00%**	**0.00%**	NA	0.00%	62.10%	NA	NA	NA	NA	NA	NA
	*P*${}_{\text{het}}^{\text{b}}$	0.753	0.677	NA	0.211	0.023	NA	NA	NA	NA	NA	NA
Methionine (μmol/L) (*N* = 6,263/7,179)	ES	−**0.25**	−0.01	−0.03	NA	NA	NA	0.94	1	0.77	0.76	0.99
	95% CI	−**0.33**, −**0.17**	−0.07, 0.06	−0.08, 0.03	NA	NA	NA	0.86, 1.03	0.91, 1.09	0.37, 1.62	0.61, 1.16	0.91, 1.21
	*P*^a^	**0.027**	0.853	0.395	NA	NA	NA	0.179	1	0.495	0.122	0.904
	*I*^2^	NA	NA	NA	NA	NA	NA	NA	NA	71.10%	65.20%	24.10%
	*P*${}_{\text{het}}^{\text{b}}$	NA	NA	NA	NA	NA	NA	NA	NA	0.063	0.09	0.251

a *P-value of the Z–test for odds ration test*.

b P-value of the Q–test for heterogeneity test

* The ES is SMD, the P^a^ is P_smd.._

** The ES is OR, the P^a^ is P_or._

The bolds pointed to subgroups that had leading to I^2^ largely decreased.

*NA, not available; US, United States; ES, effect size; 95% CI, 95% confidence interval; HPLC, high performance liquid chromatography; RIA, radioimmunoassay; CLIA, chemiluminescence immunoassay*.

#### Vitamin B6

The effect of serum B6 is presented in Figure 2 [SMD −0.28, 95%CI (−0.53, −0.02), *p* = 0.001]. Subgroup analysis by region showed that the concentration of serum B6 of LC patients was lower than the controls in Europe [SMD −0.32, 95%CI (−0.41, −0.23), *p* = 0.001] and Asia [SMD −0.38, 95% CI (−0.54, −0.23), *p* = 0.001], but not in the US [SMD −0.04, 95%CI (−0.09, 0.02), *p* = 0.207]. By gender, serum B6 level was lower in male patients than controls [OR 0.70, 95%CI (0.56, 0.87), *p* = 0.002], but not in females [OR 1.08, 95%CI (0.98, 1.21), *p* = 0.152]. By smoking status, the difference was especially significant in former smokers [OR 0.68, 95%CI (0.59, 0.78), *p* = 0.001] and current smokers [OR 0.76, 95%CI (0.62, 0.95), *p* = 0.013], but was not significant in never smokers [OR 0.95, 95%CI (0.36, 2.48), *p* = 0.910] (Table 3).

#### Vitamin B12

No significant correlation was observed between serum B12 levels and lung cancer in the overall population [SMD −0.09, 95% CI (−0.27, 0.09), *p* = 0.202] (Figure 2). Subgroup analysis showed no correlation in the Europe [SMD 0.04, 95% CI (−0.04, 0.12), *p* = 0.304] nor Asia [SMD −0.23, 95% CI (−0.46, 0.01), *p* = 0.055]. We did not observe association by method or smoking status (Table 3).

#### Homocysteine (Hcy)

Serum Hcy level of LC patients was higher than the controls in the overall population [SMD 0.41, 95% CI (0.24, 0.58), *p* = 0.001] (Figure 2). Further analysis by region showed that serum Hcy was significantly different between LC patients and the controls in both Europe [SMD 0.16, 95% CI (0.09, 0.24), *p* = 0.001] and Asia [SMD 0.45, 95% CI (0.32, 0.59), *p* = 0.001]. By method, serum Hcy was higher in patients than controls by using CLIA/RIA [SMD 0.58, 95%CI (0.32, 0.84), *p* = 0.001] and HPLC [SMD 0.34, 95%CI (0.15, 0.52), *p* = 0.001] method. No significant association was observed by smoking status (Table 3).

#### Methionine

No significant association between serum methionine levels and the risk of lung cancer was observed in the overall population [SMD −0.13, 95% CI (−0.36, 0.10), *p* = 0.001] (Figure 2). Further subgroup analysis by region showed that serum methionine was significantly different between LC patients and the controls in Europe [SMD −0.25, 95%CI (−0.33, −0.17), *p* = 0.027], but not in Asia [SMD −0.01, 95%CI (−0.07, 0.06), *p* = 0.853] nor the US [SMD −0.03, 95%CI (−0.08, 0.03), *p* = 0.395]. No association was observed by subgroup analysis according to gender or smoking status (Table 3).

### Heterogeneity and sensitivity analysis

Heterogeneity analysis showed significant heterogeneity in folate (*I*^2^ 89.4%, *P*_*het*_ 0.061), B6 (*I*^2^ 95.0%, *P*_*het*_ 0.039), B12 (*I*^2^ 32.8%, *P*_*het*_ 0.015), Hcy (*I*^2^ 61.9%, *P*_*het*_ 0.030) and methionine (*I*^2^ 96.1%, *P*_*het*_ 0.026; Figure 2). We therefore performed a sensitivity analysis to identify the source of the heterogeneity (Figures S1–S5). The results showed that the main clinical feature of the three studies (27–29) with markedly deviated 95%CI were region, thus we firstly performed a subgroup analysis according to region. Meanwhile, considering that other clinical features could influence outcome, we also performed subgroup analyses based on the strata of method, gender and smoking status. Our results indicated that region was the primary heterogeneity originator in folate, vitamin B6, vitamin B12 and Hcy, as well as method in vitamin B12, gender in folate and smoking status in folate (Table 3).

### Publication bias

**Figure 3:**
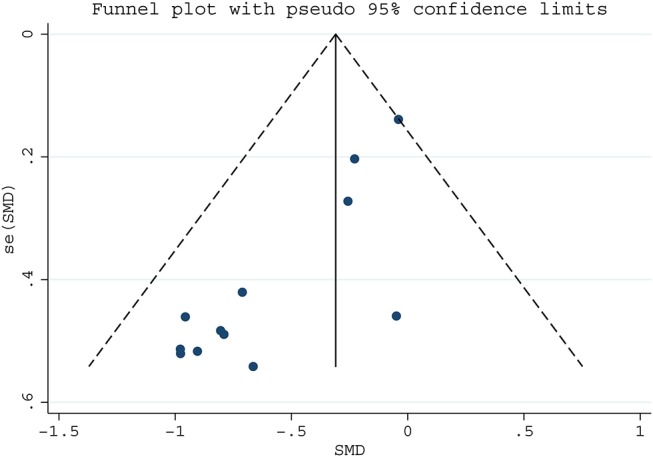
Funnel plot for assessing publication bias for serum folate and lung cancer risk.

We assessed publication bias on the articles of serum folate and LC risk by funnel plot and Egger test (Figure 3), which suggested potential publication bias (Egger's test *p* = 0.038). Then the trim-and-fill analysis was used to estimate the effect of publication bias. It was found that the results from random and fixed effects models were consistent with the original results (Table S2), indicating that there was no effect of these bias on the results, and this meta-analysis was reliable.

## Discussion

Among the factors associated with one-carbon metabolism, serum folate and vitamin B6 were found to be protective against lung cancer. Hcy was a risk factor for lung cancer. No significant correlation between B12 or methionine and the LC risk was observed.

### Association between serum OCM-related factors and LC risk

Folate acts as the core methyl donor in one-carbon metabolism, our results proved serum folate was a potential protective factor against lung carcinogenesis which was consistent with the results of other types of cancers, such as colorectal cancer (39), prostate cancer (40), and esophageal cancer (41). However, unlike a previous meta-analysis (15), our study showed statistically significant association between serum folate and the LC risk. This discrepancy may be explained by Dai study (15) included a cohort study (*n* = 15) (42) with insufficient cases and 4 case-control studies with inconsistent baseline. Although cohort study provided better evidence of causation than case-control study, the strength may be greatly reduced when the sample size is <30 with a high loss of follow-up rate.

In addition to folate, we comprehensively analyzed the association between other important factors involved in the OCM and LC risk (5, 7). For vitamin B6, we considered it as a protective factor for lung carcinogenesis and similar results were discovered in gastrointestinal cancer (43), pancreatic cancer (44), and breast cancer (45). However, a prospective cohort study (HUSK) did not find any association between serum vitamin B6 and lung cancer risk (46). We speculated that the inconsistency of these results may due to the detection of different activated forms of vitamin B6 in blood. The HUSK used 4-pyridoxic acid/pyridoxal ratio (PAr) as a new form instead of classical pyridoxal 5′-phosphate (PLP), which may be instable and has low sensitivity to detect a significant difference.

In terms of Hcy, which acts as an essential intermediate in methionine metabolic cycle. Our findings suggest that serum Hcy could contribute to lung cancer and similar results were reported in all types of cancer in a previous meta-analysis (47). However, some studies did not observe the significant association (22, 26), which may be explained by these studies restricted to the older smoking men whose physical decline (or the accumulation of unhealthy habits) belying the role of Hcy.

Regarding vitamin B12 and methionine, we did not find an associated with LC risk. For vitamin B12, some studies argued it was associated with higher mortality of LC cases (36, 48). It is possible that vitamin B12 plays different roles in pathogenesis and prognosis. As to methionine, our findings were consistent with the LC3 study (17), but the subgroup analysis (only from the EPCI study) (26) showed that serum methionine were inversely associated with LC risk, which draws speculation that methionine protects Europeans from LC rather than other populations.

Particularly, subgroup analysis results showed that the significant association between folate, vitamin B6, Hcy and LC risk was detected in Asia and Europe, but not in the US. The null result may due to only a small number of studies from the US were included (17, 26). Thus, further researches from more regions would be needed. Moreover, it was speculated that the mediating effects of OCM factors of LC would change in different regions (13, 49). Secondly, folate and vitamin B6 were found protective in ever smokers (former and current smokers) but not significant in never smokers, which might be related to the nitrites in tobacco that could obstruct the function of B vitamins or decrease its blood values (23, 27, 50, 51). Thirdly, the significant association of folate and vitamin B6 with LC was in males indicates the function of regulating androgen signaling by the key enzymes of one-carbon metabolism (9, 14). Lastly, after subgroup analysis by different detecting methods, we found no difference of the association.

### Strengths and limitations

This is the first comprehensive and systematic evaluation of the relationship between serum folate, vitamin B6, vitamin B12, homocysteine, and methionine (OCM-related factors) and the LC risk. Previous meta-analyses focused only on the association of one or two OCM factors with LC or all types of cancer risk. Moreover, we investigated blood values instead of intake level, which can better reflect the association after the factors absorbed and metabolized in the body. In addition, this study included more than 8,000 lung cancer patients who participated in very recent studies and analyzed by different subgroups, which reduced selection bias and corrected some errors in previous meta-analysis (15).

There were some limitations as well. First, although most of the original studies adjusted for variables, such as BMI, education, alcohol intake, tobacco exposure, and sex and we assessed potential sources of heterogeneity (region, gender, smoking status and method) through sensitivity analysis and subgroup analyses, we cannot completely rule out possible effects of unknown factors, such as pathology and clinical stages. Second, some epidemiological studies have shown that OCM related factors are potentially interrelated (30, 37), so we are expected to further analyze the synergistic effects of two or more factors or the dose-response on LC risk based on more data. Due to the lack of available cohort studies and RCT studies, we only included case-control studies and nested case-control studies. Eleven case-control studies might provide less certain evidence of causation, but there were three nested case-control studies with large sample size which provided more reliable evidence. The included studies were well-suited for meta-analysis as indicated by quality assessment, especially eight were high-scoring studies.

## Conclusions

This study showed that among OCM-related factors, serum folate and vitamin B6 could function as protective factors against lung carcinogenesis but that serum homocysteine could contribute to lung cancer risk. No significant association was observed between vitamin B12 or methionine and LC risk. Our findings were significant in Europe and Asia, former and current smokers, and males. Further research is needed to validate these results in other populations.

## Author contributions

JY and HL conceived and designed the study. JY, HL, and HD took full responsibility for data collecting and accuracy. JY and HL performed the meta-analysis, systematic review and drafted the manuscript. ZW helped revise the manuscript.

### Conflict of interest statement

The authors declare that the research was conducted in the absence of any commercial or financial relationships that could be construed as a potential conflict of interest.
