# Supplementary material for: Association of One-Carbon Metabolism-Related Vitamins (Folate, B6, B12), Homocysteine and Methionine With the Risk of Lung Cancer: Systematic Review and Meta-Analysis
Source: Front Oncol. 2018 Oct 31;8:493. doi: 10.3389/fonc.2018.00493 (PMC6220054; doi:10.3389/fonc.2018.00493)
Supplement: Supplementary file 1 [file Data_Sheet_1.docx]

**SUPPLEMENTARY MATERIALS**

**Table of Contents**

**Figure S1-5: Sensitivity analysis of OCM related factors** **2-4**

Folate 2

Vitamin B62

Vitamin B123

Homocysteine3

Methionine4

**Table S1: Score of quality assessment5**

Table S2: **Trim and fill method (for folate) 6**

**Supplement Appendix A：The Search Strategy for PubMed 7-8**

Figure S1: **Meta-sensitivity analysis of folate (vitamin B9)**

**
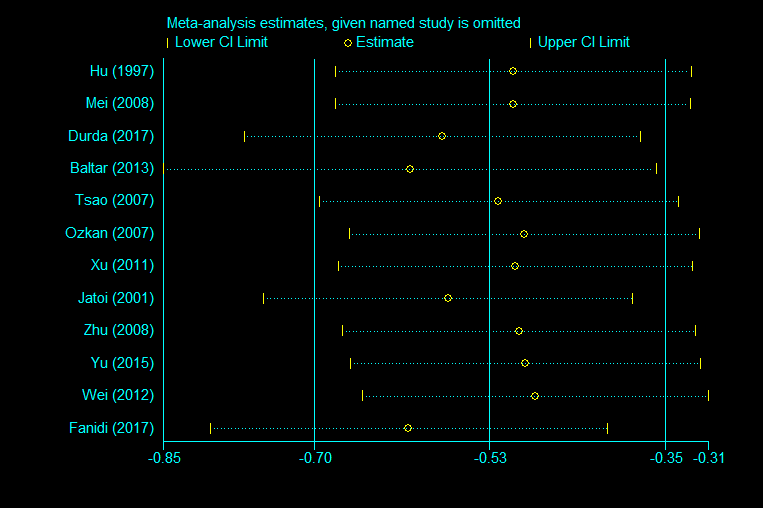
**

Figure S2: **Meta-sensitivity analysis of vitamin B6**

**
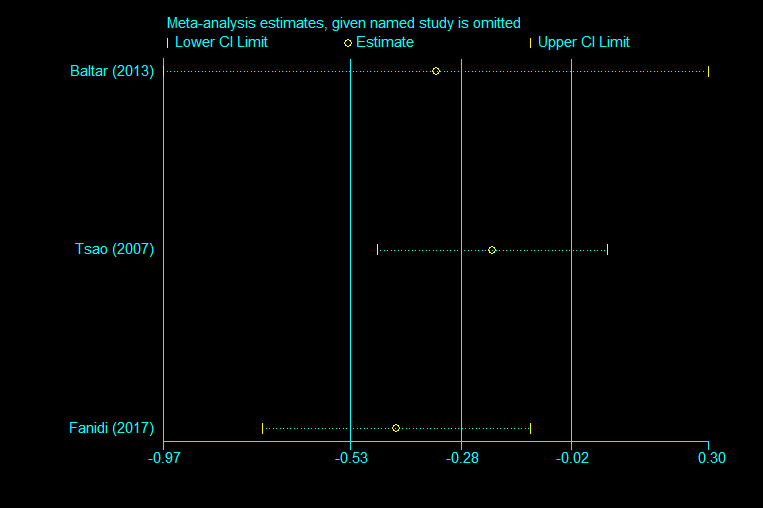
**

Figure S3: **Meta-sensitivity analysis of vitamin B12**


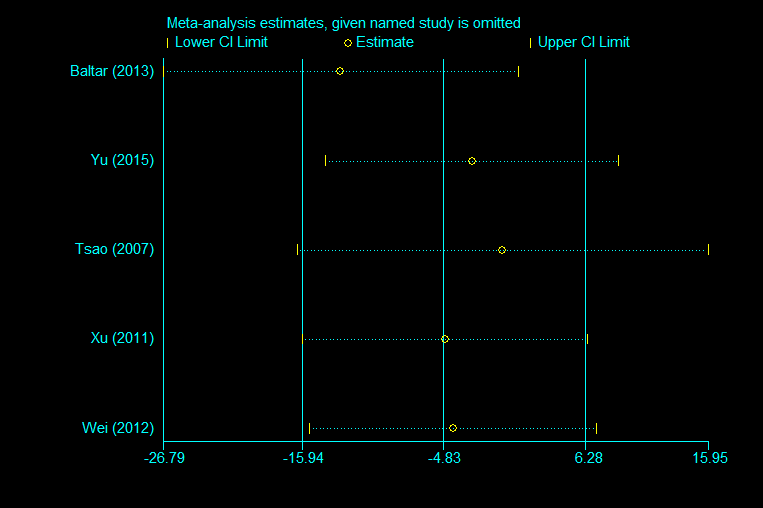


Figure S4: **Meta-sensitivity analysis of homocysteine**


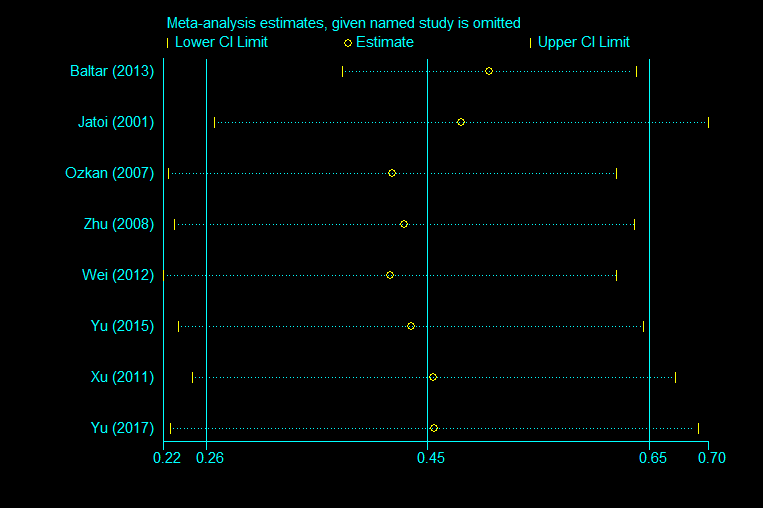


Figure S5: **Meta-sensitivity analysis of methionine**


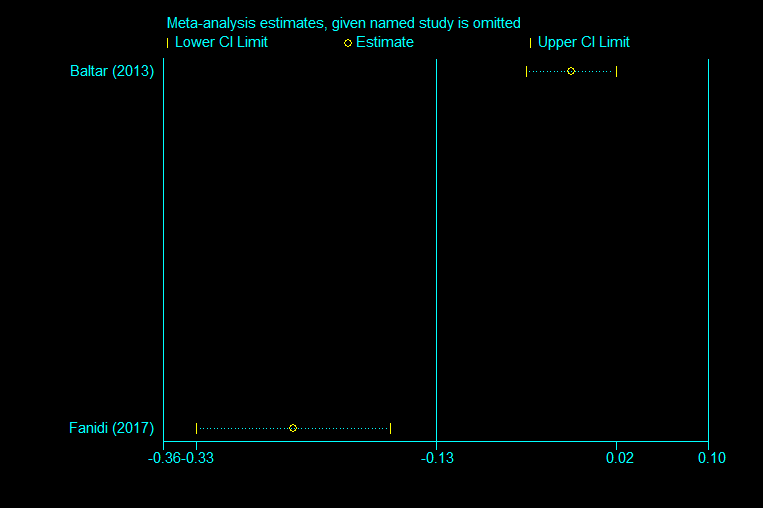


| Table S1: **Score of quality assessment** |  |
| --- | --- |
| **NEWCASTLE - OTTAWA QUALITY ASSESSMENT SCALE CASE CONTROL STUDIES** |  |
| **Selection** | **Score** |
| 1) Is the case definition adequate? |  |
| a) yes, with independent validation | **🟑** |
| b) yes, eg record linkage or based on self-reports |  |
| c) no description |  |
| 2) Representativeness of the cases |  |
| a) consecutive or obviously representative series of cases | **🟑** |
| b) potential for selection biases or not stated |  |
| 3) Selection of Controls |  |
| a) community controls | **🟑** |
| b) hospital controls |  |
| c) no description |  |
| 4) Definition of Controls |  |
| a) no history of disease (endpoint) | **🟑** |
| b) no description of source |  |
| **Comparability** |  |
| 1) Comparability of cases and controls on the basis of the design or analysis |  |
| a) study controls for _______________ (Select the most important factor.) | **🟑** |
| b) study controls for any additional factor (These criteria could be modified to indicate specific control for a second important factor.) | **🟑** |
| **Exposure** |  |
| 1) Ascertainment of exposure |  |
| a) secure record (eg surgical records) | **🟑** |
| b) structured interview where blind to case/control status | **🟑** |
| c) interview not blinded to case/control status |  |
| d) written self-report or medical record only |  |
| e) no description |  |
| 2) Same method of ascertainment for cases and controls |  |
| a) yes | **🟑** |
| b) no |  |
| 3) Non-Response rate |  |
| a) same rate for both groups | **🟑** |
| b) non-respondents described |  |
| c) rate different and no designation |  |
| Note: A study can be awarded a maximum of one star for each numbered item within the Selection and Exposure categories. A maximum of two stars can be given for Comparability. |  |

Table S2: **Trim and fill method (for folate)**

**Meta-analysis**

|  | Pooled | 95% CI | | Asymptotic | | No. of studies |
| --- | --- | --- | --- | --- | --- | --- |
| Method | Est | Lower | Upper | z_value | p_value |  |
| Fixed | -0.310 | -0.483 | -0.136 | -3.498 | 0.000 | 12 |
| Random | -0.415 | -0.640 | -0.189 | -3.595 | 0.000 |  |

**Filled Meta-analysis**

|  | Pooled | 95% CI | | Asymptotic | | No. of studies |
| --- | --- | --- | --- | --- | --- | --- |
| Method | Est | Lower | Upper | z_value | p_value |  |
| Fixed | -0.310 | -0.483 | -0.136 | -3.498 | 0.000 | 12 |
| Random | -0.415 | -0.640 | -0.189 | -3.595 | 0.000 |  |

*Explanation: By trim and fill method, both the results of fixed and random effects model are just the same with the original results*

Supplement Appendix A：**The Search Strategy for PubMed Comprised the Following**

1. "Lung Neoplasms"[Mesh]:

search ((((((((((((((((((Pulmonary Neoplasms[Title/Abstract]) OR Neoplasms, Lung[Title/Abstract]) OR Lung Neoplasm[Title/Abstract]) OR Neoplasm, Lung[Title/Abstract]) OR Neoplasms, Pulmonary[Title/Abstract]) OR Neoplasm, Pulmonary[Title/Abstract]) OR Pulmonary Neoplasm[Title/Abstract]) OR Lung Cancer[Title/Abstract]) OR Cancer, Lung[Title/Abstract]) OR Cancers, Lung[Title/Abstract]) OR Lung Cancers[Title/Abstract]) OR Pulmonary Cancer[Title/Abstract]) OR Cancer, Pulmonary[Title/Abstract]) OR Cancers, Pulmonary[Title/Abstract]) OR Pulmonary Cancers[Title/Abstract]) OR Cancer of the Lung[Title/Abstract]) OR Cancer of Lung[Title/Abstract])) OR "Lung Neoplasms"[Mesh]

1. "Vitamin B6"[Mesh]：

search ((((Vitamin B 6[MeSH]) OR Vitamin B6[Title/Abstract]) OR Phosphate, Pyridoxal[Title/Abstract]) OR Pyridoxal-P[Title/Abstract]) OR Pyridoxal 5-Phosphate[Title/Abstract]

1. "Vitamin B9"[Mesh]：

search (((((((((((((((Vitamin M[Title/Abstract]) OR Vitamin B9[Title/Abstract]) OR B9, Vitamin[Title/Abstract]) OR Pteroylglutamic Acid[Title/Abstract]) OR Folic Acid, Monopotassium Salt[Title/Abstract]) OR Folic Acid, Monosodium Salt[Title/Abstract]) OR Folic Acid, Sodium Salt[Title/Abstract]) OR Folic Acid, Calcium Salt (1:1)[Title/Abstract]) OR Folic Acid, Potassium Salt[Title/Abstract]) OR Folic Acid, (DL)-Isomer[Title/Abstract]) OR Folvite[Title/Abstract]) OR Folate[Title/Abstract]) OR Folic Acid, (D)-Isomer[Title/Abstract]) OR Folacin[Title/Abstract])) OR "Folic Acid"[Mesh]

1. "Vitamin B12"[Mesh]:

search (((((((B 12, Vitamin[Title/Abstract]) OR Vitamin B12[Title/Abstract]) OR B12, Vitamin[Title/Abstract]) OR Cyanocobalamin[Title/Abstract]) OR Cobalamins[Title/Abstract]) OR Cobalamin[Title/Abstract]) OR Eritron[Title/Abstract]) OR "Vitamin B 12"[Mesh]

1. "Homocysteine"[Mesh]：

search ((((((2-amino-4-mercaptobutyric acid[Title/Abstract]) OR 2 amino 4 mercaptobutyric acid[Title/Abstract]) OR Homocysteine, L-Isomer[Title/Abstract]) OR Homocysteine, L Isomer[Title/Abstract]) OR L-Isomer Homocysteine[Title/Abstract])) OR "Homocysteine"[Mesh]

1. "Methionine"[Mesh]：

search (((((((("Methionine"[Mesh]) OR Methionine, L-Isomer[Title/Abstract]) OR L-Isomer Methionine[Title/Abstract]) OR Methionine, L Isomer[Title/Abstract]) OR L-Methionine[Title/Abstract]) OR Pedameth[Title/Abstract]) OR Liquimeth[Title/Abstract]))

1. (2 or 3 or 4 or 5 or 6)
2. 1 and 7
